# A wide-field micro-computed tomography detector: micron resolution at half-centimetre scale

**DOI:** 10.1107/S160057752101287X

**Published:** 2022-01-19

**Authors:** Maksim A. Yakovlev, Daniel J. Vanselow, Mee Siing Ngu, Carolyn R. Zaino, Spencer R. Katz, Yifu Ding, Dula Parkinson, Steve Yuxin Wang, Khai Chung Ang, Patrick La Riviere, Keith C. Cheng

**Affiliations:** a Department of Pathology, Penn State College of Medicine, Hershey, Pennsylvania, USA; b The Jake Gittlen Laboratories for Cancer Research, Penn State College of Medicine, Hershey, Pennsylvania, USA; c Biomedical Sciences PhD Program, Penn State College of Medicine, Hershey, Pennsylvania, USA; d Medical Scientist Training Program, Penn State College of Medicine, Hershey, Pennsylvania, USA; e Advanced Light Source, Lawrence Berkeley National Laboratory, Berkeley, California, USA; f Mobile Imaging Innovations, Inc., Palatine, Illinois, USA; g Penn State Zebrafish Functional Genomics Core, Penn State College of Medicine, Hershey, Pennsylvania, USA; h Department of Radiology, The University of Chicago, Chicago, USA

**Keywords:** synchrotron micro-CT, high resolution, large field of view, X-ray optics, histotomography

## Abstract

Ideal three-dimensional imaging of complex samples made up of micron-scale structures extending over mm to cm, such as biological tissues, requires both wide field of view and high resolution. For existing optics and detectors used for micro-CT (computed tomography) imaging, sub-micron pixel resolution can only be achieved for fields of view of <2 mm. This article presents a unique detector system with a 6 mm field-of-view image circle and 0.5 µm pixel size that can be used in micro-CT units utilizing both synchrotron and commercial X-ray sources. A resolution-test pattern with linear microstructures and whole adult *Daphnia magna* were imaged at beamline 8.3.2 of the Berkeley Advanced Light Source. Volumes of 10000 × 10000 × 7096 isotropic 0.5 µm voxels were reconstructed over a 5.0 mm × 3.5 mm field of view. Measurements in the projection domain confirmed a 0.90 µm measured spatial resolution that is largely Nyquist-limited. This unprecedented combination of field of view and resolution dramatically reduces the need for sectional scans and computational stitching for large samples, ultimately offering the means to elucidate changes in tissue and cellular morphology in the context of larger, whole, intact model organisms and specimens. This system is also anticipated to benefit micro-CT imaging in materials science, microelectronics, agricultural science and biomedical engineering.

## Abstract 2

A custom wide-field lens and a new-generation megapixel camera enabled micro-CT scanning over a 3.5 mm × 5 mm field of view at 1 µm resolution/0.5 µm pixel size at Lawrence Berkeley Laboratory’s Advanced Light Source and Argonne National Laboratory’s Advanced Photon Source using a phantom with micron-scale features. This novel combination of resolution and field of view was designed for broad applicability to any setting in which micron-scale structures need to be characterized comprehensively in three dimensions over mm to cm.

## 1. Introduction

### 1.1. Challenges in mesoscale imaging and micro-CT

Micro-computed tomography (micro-CT) is rapidly becoming a valuable imaging technique for applications where the generation of high-resolution, isotropic, three-dimensional data sets is essential for visualization and both qualitative and quantitative phenotyping (Weinhardt *et al.*, 2018 ▸; Hur *et al.*, 2018 ▸; Babaei *et al.*, 2016 ▸; Seo *et al.*, 2015 ▸). In comparison, three-dimensional reconstructions at nm-scale resolution can be accomplished using serial electron microscopy at the cost of field of view and data handling; acquiring and analyzing mm-scale samples is a physically and computationally protracted process (Hildebrand *et al.*, 2017 ▸). Lower-resolution imaging methods like magnetic resonance imaging generate proportionately smaller data sets for the same field of view, suitable for scanning much larger samples, but are limited to tens to hundreds of microns in resolution (Dodd *et al.*, 1999 ▸; Edlow *et al.*, 2019 ▸). Micro-CT bridges the gap between resolution, field of view and analytical feasibility by offering isotropic micron-scale resolution for mm- to cm-scale samples (Mizutani *et al.*, 2013 ▸; Ding *et al.*, 2019 ▸). There is growing demand for micro-CT imaging in the disease model research community (Cheng *et al.* 2011 ▸; Weinhardt *et al.*, 2018 ▸), potentially for anchoring spatially resolved large-scale molecular analyses. Large-field, high-resolution images are needed, however, to pursue the full range of quantitative differentiation of cellular and architectural tissue features that are currently indistinguishable using traditional histological (two-dimensional) analysis. Commercial micro-CT scanners available from companies such as Zeiss, Siemens and Bruker use either commercial flat-panel detectors or microscope objective lenses with field-of-view-to-resolution ratios of about 1000, compared with 10000 in this work.

### 1.2. Advances in synchrotron-based micro-CT

Many efforts to improve the resolution and field of view of micro-CT utilize large detector arrays and non-traditional image acquisition/reconstruction techniques. Synchrotron sources’ tunable, brilliant, monochromatic and parallel X-rays additionally provide an opportunity for the development of improved optics systems such as the one demonstrated here. Image acquisition and data processing techniques such as phase-contrast imaging for unstained soft tissue, spiral-CT acquisition for increasing field of view, dual-energy scanning for single stains and simultaneous use of multiple stains, and post-acquisition software-based processing tools such as distortion correction have allowed micro-CT imaging to mitigate the limitations in detector technologies (Barbone *et al.*, 2021 ▸; Martins de Souza e Silva *et al.*, 2017 ▸; Sawall *et al.*, 2012 ▸; Vo *et al.*, 2015 ▸; Pelt & Parkinson, 2018 ▸). Recently, a phase-contrast application using a 2000 × 2000 PCO edge 4.2 CLHS camera was able to achieve cm-scale whole-organ imaging at 25 µm voxel size with the capability of zooming in to 1.4 µm voxel size in areas of interest. This was achieved through a combination of the increased coherence and brilliance of a fourth-generation synchrotron, a custom sample mounting and image acquisition approach to extend the dynamic range of two detectors, and stitching/feathering of reconstructions (Walsh *et al.*, 2021 ▸). Alternatively, the direct development of higher-resolution optics systems allows implementation in any of the previously mentioned applications; one research group adapted a commercial 36 Mpixel digital single-lens reflex camera for visualizing whole secondary pulmonary lobules in large human lung specimens, resulting in a 40.6 mm wide × 15.1 mm high field of view with 3.07 µm pixel size without any advanced acquisition techniques (Umetani *et al.*, 2020 ▸). Despite these advances in detectors and acquisition/reconstruction mechanisms, there is still a fundamental trade-off between resolution and field of view in micro-CT systems; cm-scale field-of-view applications are generally limited to a multiple-micron resolution, while the detection of micron-scale features is normally limited to sub-mm fields of view (Bailey *et al.*, 2017 ▸; Kc *et al.*, 2019 ▸; Shearing *et al.*, 2011 ▸). This poses a challenge to studies seeking to thoroughly investigate large, complex samples at high resolution, ranging from disease models (*e.g.* developing mice, zebrafish, *Daphnia*) to micro-circuits in electronic components and fuel spray systems (Wong *et al.*, 2012 ▸; Ding *et al.*, 2019 ▸; De Samber *et al.*, 2008 ▸; Lall & Wei, 2015 ▸; Tekawade *et al.*, 2019 ▸).

### 1.3. Development of the wide-field detector

Most detector systems for micro-CT applications use objective lenses designed for cameras with sensors with up to 5 million pixels. These systems have been limited by both the availability of scientific CMOS sensors and field-of-view limitations of magnifying optics. The rapid development of CMOS sensors for both consumer and commercial imaging applications led to the availability of CMOS sensors with over 100 million pixels with excellent noise performance at reasonable cost levels. The key to making use of these large-format sensors in a detector system with micron-scale resolution is an objective lens with large field of view, low magnification, high numerical aperture, low distortion and high throughput for the scintillator emission spectrum. Since no commercial lens currently meets these requirements, we collaborated with Mobile Imaging Innovations, Inc., to develop a custom objective lens matching an Advanced Photo System-size CMOS sensor. In our prototype we used a 71 Mpixel thermo-electrically cooled CMOS camera in 10 000 × 7096 pixel format and 3.1 µm pixel size. The custom objective lens provides 6.2× magnification and switchable numerical aperture of 0.6/0.75, with diffraction-limited resolution across the entire field at a numerical aperture of 0.6. Effectively this objective lens provides the field of view of a conventional 4× microscope objective lens at the resolution of a 40× objective. Our custom detector represents a factor of 100× gain in field of view and 1000× gain in scan volume compared with commercial objective lenses, enabling far more extensive studies of complex material science samples, engineered devices and biological systems of mm to cm scale. Here, we present measurements of this detector’s resolution and field of view based on data acquired at the Berkeley Advanced Light Source (ALS) and the Argonne Advanced Photon Source (APS) synchrotrons using a test phantom that includes a Siemens star and parallel lines with µm line width, and two biological species, *Daphnia magna* and *Danio rerio* (zebrafish). This system sets a new benchmark for large-field resolution in synchrotron and tabletop micro-CT implementations.

## 2. Materials and methods

### 2.1. Detector design

All experiments reported in this article used LuAG:Ce scintillators with a 15 mm diameter and 100 µm thickness from Epic Crystal, polished on both sides, with anti-reflective coating applied on the exit side. Our custom objective lens was developed by Mobile Imaging Innovations, Inc., and CMOS cameras were acquired from Vision Systems Technology. The camera used in this experiment is an Advanced Photo System-sized CMOS sensor (CHR70M) with 71 million pixels arranged in 10 000 × 7096 pixel format with 3.1 µm pixel size, resulting in a 5 mm × 3.5 mm field of view at 6.2× magnification (Vieworks VP-71MC produced by Vision Systems Technology). The camera operates at 4.2 frames per second, has a native image depth of 12 bits and uses a Camera Link interface. Although the camera’s detector was not a designated scientific CMOS sensor, we used a grade-A chip with three-stage thermal electric cooling to significantly improve the noise performance and reduce the number of bad pixels. The resulting performance approaches that of a scientific-grade CMOS sensor.

The Crytur data sheet for LuAG:Ce scintillators gives a photon yield of 25 photons keV^−1^, meaning that each 5 keV photon would be converted to 125 photons of visible light. At a numerical aperture (NA) of 0.75 for the objective lens, the collection throughput, as calculated by [1 − cos(half angle)]/2, is 0.17. The quantum efficiency of the CHR70M sensor at 535 nm is close to 100%. Accounting for up to 10% transmission loss in the system, the total throughput is 19 electrons for each X-ray photon at 5 keV, sufficient to overcome temporal and fixed pattern noise in the sensor.

This custom lens provides 6.2× magnification with a switchable NA of 0.6/0.75. The diffraction-limited optical pixel size of 0.5 µm is achieved at an NA of 0.6 across the entire field of view and maintained when used at 0.75 with a 55% increase in throughput. The image circle achieved using this CMOS sensor was over 6 mm on the scintillator plane, creating an effective field of view of 5 mm × 3.5 mm with a pixel size of 0.5 µm. To our knowledge, the only optics with comparable resolution and field of view are projection lenses for lithography, which operate at a narrower bandwidth at much greater cost (Kawata *et al.*, 1989 ▸).

### 2.2. Animal husbandry and sample preparation

*Daphnia magna* (*D. magna*) were raised in modified artificial freshwater ‘Aachener Daphnien Medium’ or ADaM (Ebert *et al.*, 1998 ▸) at room temperature (20°C ± 1°C) under a 16 h light/8 h dark photoperiod. The animals were fed three times weekly with green microalgae (*Raphidocelis subcapitata*, previously known as *Selenastrum capricornutum*) at a concentration of 3.0 × 10^7^ cells ml^−1^ and 0.1 mg ml^−1^ of dissolved bakers’ yeast. The embedding protocol was adapted from Lin *et al.* (2018 ▸). *D. magna* were fixed in Bouin’s for 2 days at room temperature and then stained with 2% phosphotungstic acid (PTA) (in 70% ethanol) for another 2 days prior to embedding.

Zebrafish were housed in Penn State Zebrafish Functional Genomics Core recirculating aqua­ria at an average temperature of 28°C and on a 14 h light/10 h dark cycle. The fish were fed three times per day with brine shrimp and dry flake food. The wildtype zebrafish were staged according to a standard developmental staging series (Kimmel *et al.*, 1995 ▸). Zebrafish fixation and embedding were done as described by Lin *et al.* (2018 ▸). All procedures on zebrafish were approved by the Institutional Animal Care and Use Committee (IACUC) at the Pennsylvania State University College of Medicine, IDs PROTO201800300, ‘Developing Tissue-, Cell-, and Protein-specific Staining for histo-tomography, a form of X-ray Microtomography (micro-CT)’, and PRAMS201445659, ‘Groundwork for a Synchrotron MicroCT Imaging Resource for Biology (SMIRB)’.

### 2.3. Imaging parameters

**Figure 1:**
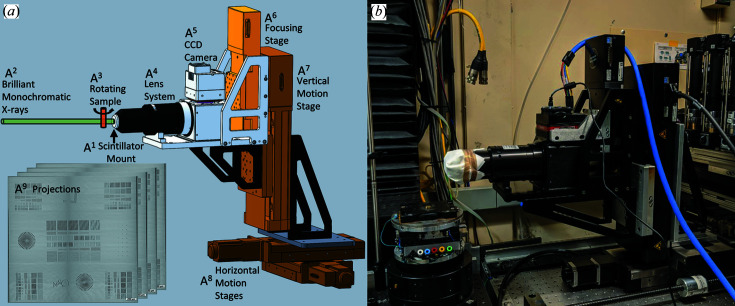
Generalized beam alignment and image acquisition setup for application in synchrotron facilities and tabletop sources. CAD design (*a*). Actual setup as assembled in the Berkeley ALS 8.3.2 beamline hutch (*b*). Our custom detector operates using a scintillator (A^1^) to convert X-rays (A^2^) passing through a sample (A^3^) into visible light. The fluorescence image is then focused by the objective lens (A^4^) onto the CMOS sensor surface (A^5^). Commercially available Newport stages (A^6^–A^8^) were utilized to adjust the camera relative to the focal point, and to translate the detector through three-dimensional space (15 cm vertical travel and 10 cm horizontal travel in any direction) for optimal alignment with any synchrotron- or tabletop-generated beam. Projections (A^9^) acquired by the detector are saved using the *Harvester* Python library and later reconstructed with the *TomoPy* toolbox in Python.

All samples were scanned at beamline 8.3.2 at the ALS at Lawrence Berkeley National Laboratory, with the exception of projection data of the phantom acquired at the Argonne APS 7-BM beamline. Alignment of the detector with the synchrotron beam was accomplished with a custom mounting system comprising two horizontal Newport MTN100PP stages, a vertical Newport IMS150PP stage, Thorlabs 90° mounts, and custom-designed milled adapters to integrate imperial and SI components (Fig. 1 ▸). ALS scan geometry included 7.6 cm between the object and scintillator after orthogonal alignment to the beam for QRM Nanobar phantom (QRM) acquisitions, and 4.3 cm for *Daphnia*. All scans of the phantom and *Daphnia* were acquired at 20 keV, as a sequence of 150 ms projections for *Daphnia* and 5000 ms for the phantom, due to the phantom scans being acquired at lower flux during the beamline’s operation on two-bunch mode. Acquisitions were made using a W/B4C multilayer monochromator with a *d*-spacing of 2 nm and 1% bandpass, allowing an energy range of 8–45 kV. The QRM Nanobar phantom was acquired as 7873 projection images. Projections were acquired over 180° and reconstructed with parallel-beam reconstruction techniques through *TomoPy* (Fig. 3). The number of projections was determined by

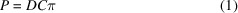

where *P* is the total number of projections, *D* is the furthest horizontal distance in pixels from the center of rotation to the edge of the sample in the field of view and *C* is the number of pixels per micron. *Daphnia* scans were acquired with ∼3000 projections each, depending on the diameter of the sample.

Projections of the phantom taken at APS and used exclusively for the data shown in Fig. 5 were acquired using similar parameters, with the following exceptions: the smallest possible source-to-scintillator distance, 3 mm, was used to reduce phase effects in the calculation of the modulation transfer function, and the highest available scanning energy of 18 kV was chosen to approach the scanning parameters at ALS.

### 2.4. Detector setup at beamline 8.3.2

Initial detector testing and validation were done at the Berkeley ALS 8.3.2 beamline to take advantage of synchrotron-beam properties over those offered by commercial tabletop sources (Fig. S1 in the supporting information): the brilliance of the synchrotron X-rays allows for shorter scan times, reducing drift artifact (Figs. S1A, SB). The high monochromaticity, tunability and coherence aspects of the synchrotron beam allowed the measurement of detector response as a function of X-ray energy, selective tuning of phase-contrast edge enhancement, and the use of conventional parallel-beam reconstruction approaches (Figs. S1C, SD). Single CT data set acquisitions took approximately 30 min at 20 keV and an object-to-scintillator distance of 7.6 cm, compared with >48 h imaging sessions under similar conditions to achieve a comparable signal-to-noise ratio (SNR) on the local tungsten NOVA 96000 source at Penn State. To facilitate alignment of the system with multiple synchrotron facilities and local tabletop X-ray sources, a motion control system with three axes of motion (10 × 10 × 15 cm) was used (Fig. 1 ▸).

### 2.5. Acquisition, reconstruction and data visualization

Image acquisition from the CMOS camera was controlled by a Python script using the *Harvester* library (https://github.com/genicam/harvesters). The stage holding the sample was set to rotate at constant velocity and trigger camera-based projection acquisition at preselected intervals. Flat-field correction, ring artifact reduction and reconstruction were performed using the *TomoPy* toolkit (Gürsoy *et al.*, 2014 ▸). Flat-field correction used either the flat image taken before or after sample scanning, depending on which time point yielded the most contrast with test projections. Reconstructions were computed using the *gridrec* algorithm (Dowd *et al.*, 1999 ▸; Rivers, 2012 ▸) with a Shepp–Logan filter. Stripe correction on the sinogram domain was performed using a combination of wavelet–Fourier filtering and Titarenko’s algorithm (Miqueles *et al.*, 2014 ▸; Münch *et al.*, 2009 ▸). Reconstructions resulted in a nominal isotropic voxel size of 0.52 µm, as estimated by the number of pixels spanning the 1.03 mm diameter of a reconstructed polyimide tube used for sample embedding. All data were visualized in *ImageJ*.

### 2.6. Resolution measurements

All visual confirmation of resolution based on demarcations present on the phantom, whether in projection or reconstruction domains, was done by visualizing data in *ImageJ* and adjusting image window/level to optimize contrast. Intensity line profiles were calculated as a vector of individual intensity values taken across indicated areas. The line spread function (LSF) was calculated as

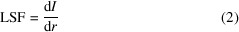

where *I* is the intensity line profile and *r* is distance. The modulation transfer function (MTF) was then calculated as the discrete normalized Fourier transform of the LSF in the direction of intensity variation of *I*.

## 3. Results

### 3.1. Characterization of field of view

**Figure 2:**
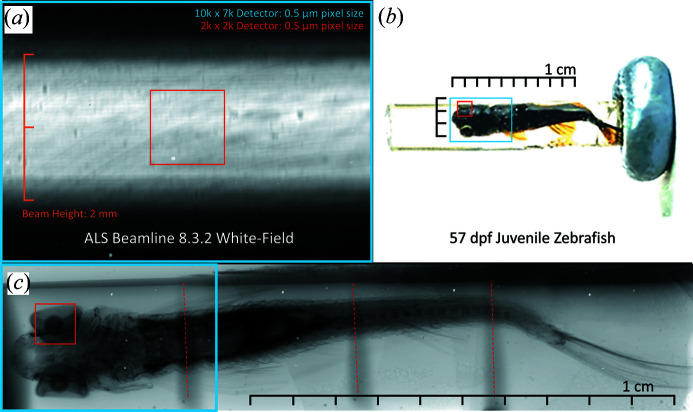
Field-of-view characterization utilizing the ALS synchrotron beam and large-scale biological sample. The entirety of the beam height at the ALS 8.3.2 beamline is encapsulated by the detector’s field of view while maintaining 0.5 µm pixel size (*a*). A 2000 × 2000 pixel field of view at the same 0.5 µm pixel size is shown in red. To illustrate the field of view in a more biologically applicable and practical sense, we present a cm-scale, juvenile, 57 day post-fertilization (d.p.f.) zebrafish embedded in resin and stained with phospho­tungstic acid (PTA) (*b*) using a local NOVA 96000 tungsten filament source to demonstrate field of view (*c*), with coverage of the same 2000 × 2000 pixel array in red and our detector’s field of view in blue. Rotating the camera by 90° makes it possible to scan in a single view horizontally and to stitch in four sections vertically, compared with approximately 500 scans on a 2000 × 2000 detector to cover the same volume. Red dotted lines indicate areas of field-of-view overlap.

Upon centering the beam with the detector, initial projection tests confirmed our detector’s field of view as larger than what is available for typical synchrotron applications [Fig. 2 ▸(*a*)]. While the detector’s 5 mm horizontal field of view was sufficiently covered by the beam, the 3.5 mm vertical range encapsulated the full beam height, allowing the acquisition of samples previously unattainable on similar setups [Fig. 2 ▸(*b*)]. The approximately 2 mm beam height at the Berkeley ALS beamline 8.3.2 for these experiments was limited by the monochromator – using longer multilayer mirrors in a redesigned monochromator would allow us to utilize the full field of view of this detector. The 8.3.2 beamline’s optics capabilities can expand the horizontal field of view of their detection system to >10 mm at a 9 µm pixel size. For comparison, the beamline’s 2000 × 2000 pixel CMOS camera set to a 0.5 µm pixel size would have a field of view of 1 mm × 1 mm, 1/17.5 of our detector’s area [Figs. 2 ▸(*a*), 2 ▸(*b*), 2 ▸(*c*)]. As a functional demonstration of the field of view, we imaged a juvenile, 57 day-old zebrafish [Fig. 2 ▸(*b*)]. A 2000 × 2000 pixel detector with an equivalent 0.5 µm pixel size would require hundreds of individually acquired and stitched scans across both the horizontal and vertical field of view to cover the same reconstructed volume as four scans on the custom detector stitched only in the axial dimension [Fig. 2 ▸(*c*)]. When performing tomography of large objects, stitching data in the axial direction is relatively straightforward and can be done after tomographic reconstruction. Stitching data in the radial direction is challenging since this must be done in the sinogram domain; small inconsistencies in the stitching or calibration of rotation axes are amplified by tomographic reconstruction and lead to artifacts in reconstructed images (Vescovi *et al.*, 2018 ▸).

### 3.2. Quantification of resolution

**Figure 3:**
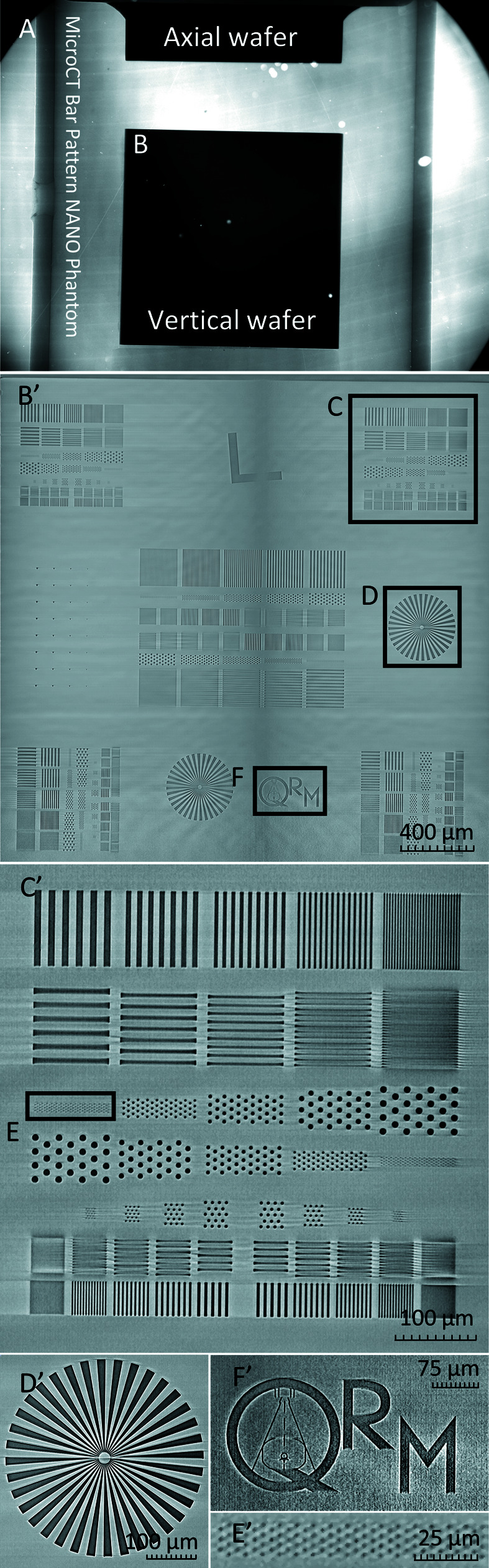
Reconstruction of QRM nanobar phantom die acquired over a single projection series. Projection from the side including both case dies (*a*). The zoomed-in elements of the reconstructed vertical die (*b*′) qualitatively demonstrate the resolution of elements of defined size. Patterns including line pairs and dots ranging from 10 µm to 1 µm (*c*′) are shown, along with a dot pattern containing elements that are 2 µm in diameter (*e*′), an etched QRM symbol (*f*′), and the reconstruction of the same Siemens star (*d*′) previously shown as a projection in Fig. S1.

Our resolution was qualified using a Nanobar phantom fabricated by QRM consisting of two perpendicular 2.96 mm^2^ silicon dies that each include sets of specified line pairs and dot patterns ranging from 10 µm to 1 µm feature size, Siemens stars converging to 1 µm at the points, and an *L* edge, encapsulated in a 5 mm-thick solid plastic (Fig. 3 ▸). All structures are etched in the silicon die with a recorded depth of 5–15 µm. The field of view offered by our custom detector enabled the capture of both chips in a single acquisition sequence [Fig. 3 ▸(*a*)].

**Figure 4:**
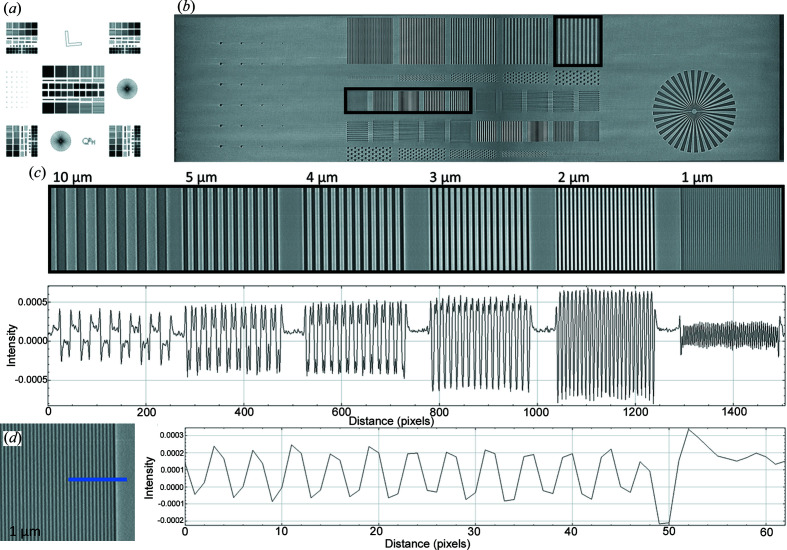
Verification of resolution utilizing a QRM bar pattern phantom. A QRM-manufactured phantom including line and point patterns, Siemens stars and an *L* edge (*a*) was scanned at the Berkeley ALS and locally reconstructed to characterize the resolution of the combined lens system and camera detector. A portion of the reconstructed phantom is presented (*b*) showcasing line pairs ranging from 10 to 1 µm in width. A line profile of the indicated regions is shown, validating resolution up to 1 µm (*c*). A smaller line profile taken across the 1 µm line pairs that highlights signal shape is shown (*d*). Signal at the edges of line pairs is enhanced by phase effects caused by the silicon–air interface. Pixel counts between line pairs confirm reconstruction at 0.5 µm, as each line of the 1 µm pattern is 2 pixels thick.

The spatial resolution of the three-dimensional reconstruction was measured from a segment of the reconstructed image that contains line pairs as the simplest method to showcase the detector’s ability to discern 1 µm features (Fig. 4 ▸). Although the detector provides a nominal resolution of 0.5 µm, the width of the smallest 1 µm line pairs is at the Nyquist-limited resolution with our presented acquisition and reconstruction pixel size of 0.5 µm (Fig. 4 ▸).

**Figure 5:**
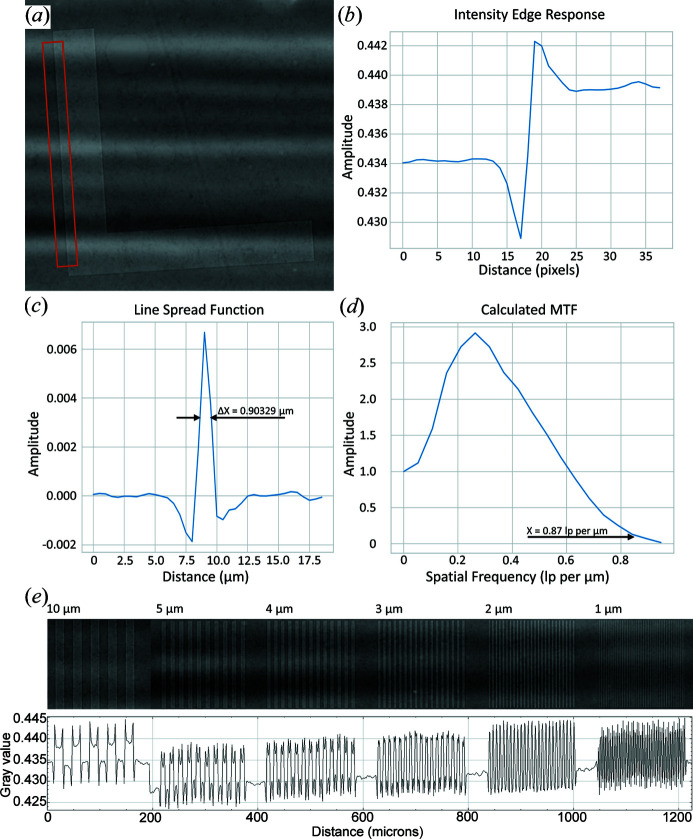
Slanted edge response and calculation of MTF in the projection domain. A 400 projection series taken at the same angle of the indicated area of the slanted *L* edge (*a*) was used to record the associated averaged intensity line profile (*b*). The LSF (*c*) was taken as the first derivative of (*b*) across pixel distance, and the MTF (*d*) was calculated as the discrete normalized Fourier transform of the portion of (*c*) covering the edge. The full width at half-maximum amplitude of the LSF was recorded at 0.90 µm (*c*), with the indicated 0.1 MTF amplitude located at 0.87 line pairs per µm (*d*). Line pairs are presented with a corresponding averaged intensity line profile indicating contrast within the 10, 5, 4, 3, 2 and 1 µm line pair groups (*e*). Edge enhancement can be visualized most easily in the intensity profile region corresponding to the 10 µm demarcations.

An intensity profile taken through these line pairs confirmed visible contrast down to a resolution of 1 µm. As an additional measurement of our resolution, the *L* edge of the Nanobar phantom was used to calculate the LSF as well as the MTF in both the reconstructed domain as well as in independently acquired projections. A total of 400 projections taken at a single angle were averaged for this measurement, acquired at the Argonne National Laboratory APS using a minimal sample-to-detector distance of approximately 3 mm to minimize potential phase effects. The line profile measurements taken under these conditions yielded the full width at half-maximum of the LSF as 0.90 µm, and a drop-off to the 0.1 amplitude of the MTF at 0.87 line pairs per µm (Fig. 5 ▸).

These results are consistent with our ability to qualitatively discern 1 µm features on the phantom at a 0.5 µm pixel size. Overall, we achieve a pixel size of 0.5 µm and a spatial resolution of 0.90 µm as measured at an NA of 0.6 across a 5 mm × 3.5 mm field of view, and these specifications are maintained when used at an NA of 0.75 with a 55% increase in throughput. The effects of tomographic reconstruction, although boosting the signal through slices of the phantom used for analysis, increased the full width at half-maximum amplitude of the LSF to 1.58 µm and the 0.1 MTF amplitude to 0.28 line pairs per µm (Fig. S2). This slight loss in resolution in the reconstruction domain is likely due to the various interpolations and filters used during tomographic reconstruction and post-reconstruction interpolation due to rotating in each of three orthogonal planes to bring the test pattern *en face*.

### 3.3. Biological applications in *Daphnia*

**Figure 6:**
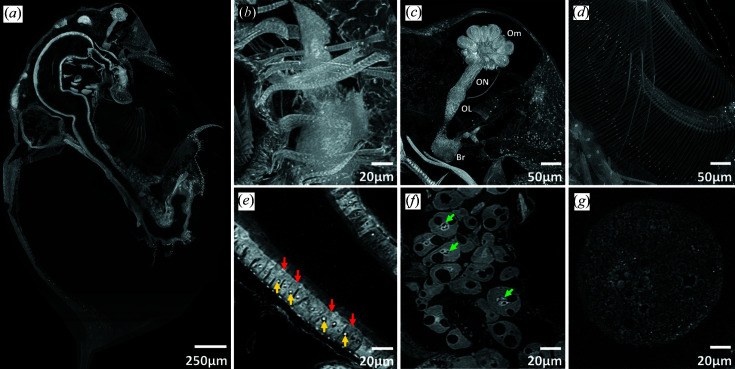
Adult *D. magna* stained with PTA showing sub-cellular resolution for different cell types and structures (*a*). Cell types and structures that can be visualized by customizing various stack thicknesses included: muscle striations (about 2 µm) on the labral muscles (*b*), ommatidia (Om), optic nerves (ON), optic lobe (OL) and brain (Br) (*c*), setae of the filter plates (*d*), nucleoli (yellow arrows, ∼2 µm diameter) in the gut epithelial cells (red arrows) (*e*), nucleoli (green arrows) in the fat cells (*f*) and yolk globules (*g*) in a developing embryo. Panels (*a*) to (*c*) were generated from maximum intensity projections (MIP) of 50 slices, panel (*d*) from an MIP of 100 slices to visualize thicker three-dimensional structures and panels (*e*) to (*g*) from an MIP of ten slices. An MIP of ten slices is equivalent to a 5 µm-thick histology slide.

*D. magna* is a freshwater crustacean that plays a significant role in the food web, has relatively high sensitivity to environmental contaminants, and has been commonly used as a model organism for toxicity testing and environmental biomonitoring for over half a century (Anderson, 1944 ▸; Environmental Protection Agency, 2002 ▸, 2016 ▸; OECD, 2001 ▸, 2004 ▸, 2012 ▸). To demonstrate the field of view and resolution of our detector system, we scanned an entire adult wild-type *D. magna* at the ALS. The resulting three-dimensional reconstructions allow virtual, histology-like ‘sections’ in any plane and three-dimensional rendering at user-defined scales to reveal micro-anatomic features of organ systems, tissues and cells in the context of the entire organism at sub-cellular resolution (Fig. 6 ▸). Details of thicker tissues or organs, such as the connections between ommatidia, optic nerves, optic lobe and brain [Fig. 6 ▸(*c*)], and the long setae (hair-like structures) of filter plates [Fig. 6 ▸(*d*)], can be visualized through customizing maximum intensity projection (MIP) thicknesses. Reconstructed voxel sizes of 0.5 µm allow visualization of cellular structures, such as nucleoli (about 2 µm in diameter) of gut epithelial cells [Fig. 6 ▸(*e*)], nucleoli of fat cells [Fig. 6 ▸(*f*)] and yolk globules in a developing embryo [Fig. 6 ▸(*g*)]. Visualizing and characterizing changes in these structures are important because they play important roles in development, reproduction and energy processing (Fryer, 1991 ▸; Quaglia *et al.*, 1976 ▸; Zaffagnini & Zeni, 1986 ▸; Colbourne *et al.*, 2011 ▸).

The ability to interrogate the cellular and organ-based structures throughout entire organisms is essential for characterization of toxicity response, development and morphological effects. To exemplify the detection of cellular change, we imaged an abnormal wild-type *D. magna* where the compound eye had developed outside the carapace (Fig. S3). Micro-CT imaging detected the deformities of the ommatidia (less than the usual 22 ommatidia), longer optic nerves and absence of an optic lobe (Fig. S3B). Moreover, we have detected cellular changes (vacuolation and sloughing of epithelial cells) in the gut (Fig. S3C) which would potentially go unnoticed using an imaging technology that does not consider the entire specimen at cellular detail, such as histology, electron microscopy or micro-CT that compromises on either field of view or resolution. Broad availability of a detector with the present characteristics would enable whole-organism imaging to be used in a comprehensive approach to systematic characterization of three-dimensional morphological and cellular changes, not only in *Daphnia* research but for other mesoscale model organisms.

## 4. Discussion

### 4.1. Detector capabilities and nonconventional applications

The compromise between resolution and field of view is a challenge for imaging that has not been fully addressed for mesoscale imaging applications by either synchrotron facilities or commercial companies. Current synchrotron-based high-resolution micro-CT systems rely on microscope objective lenses to magnify scintillated images or flat-panel detectors developed for radiology. The resulting resolution to field of view ratio is limited to microscopy standards, about 1/1000. In most sub-micron-resolution applications, the field of view is typically limited to 1 mm. Our custom detector provides a 1 µm-scale resolution over a 5 mm field of view, enabling unique imaging applications of large samples in single field-of-view acquisitions. Additionally, even larger samples falling outside the capabilities of the detector can be scanned by further expanding the wide field of view with spiral micro-CT acquisition (Pelt & Parkinson, 2018 ▸). Furthermore, mosaic spiral-CT, which takes multiple spiral-CT passes, can be implemented to significantly expand the effective field of view beyond that of the detector without having to spend resources to stitch a large number of individual small scans in both horizontal and vertical directions, which can propagate stitching errors that create artifacts in the reconstructed three-dimensional images. For example, to accommodate samples about twice as wide as the camera’s field of view horizontally and many times larger vertically, the detector can first be positioned to cover the left half of the object, and a spiral CT sequence can be acquired throughout its full length. Then a second spiral CT data set is acquired over the right half. Two-dimensional projections from the two data sets acquired at each angle and vertical position can then be stitched pairwise to form larger two-dimensional projections that span the entire width and length of the object. A mosaic spiral acquisition approach utilized on this detector will enable a new range of applications for imaging larger biological specimens such as intact mouse embryos and excised brains, adult zebrafish and clinical samples, as well as other micro-CT use cases centered on non-biological samples, including those from geology, materials science, electronics and other industries. Alternatively, the field of view and resolution combination afforded by the detector can be leveraged to increase throughput of high-resolution imaging of smaller specimens by multiplexing multiple samples in a single acquisition and reconstruction.

### 4.2. Implications for model system research

Properties such as cellular and organellar shape and volume, anatomical locations, intercellular relationships, physiology, environment and state of health are reflected in morphological changes on the micron scale whose meaning is apparent in the context of the whole organism. Using our detector system, we demonstrate visualization and characterization of non-targeted cellular changes enabled by whole-organism imaging of *D. magna*, in a single acquisition. We envision the systematic characterization of three-dimensional morphological and cellular changes as part of a more comprehensive approach to the evaluation of environmentally and genetically induced morphological changes. Looking forward, comprehensive phenotyping of larger disease and toxicological model organisms, such as adult zebrafish, mouse embryos and *Xenopus*, for quantitative measurements of tissue architecture and cellular features will contribute to the systematic understanding of pathological change.

## 5. Conclusions and future work

The applicability of micro-CT to any field is dependent on accommodating both typical sample sizes and resolving components pertinent to the research pursued. In the case of biology, there is a need to accommodate the full size of the smallest vertebrate models and to resolve all of their cell types and their common adaptations in health and disease. All the tests that we have done confirm our claimed/expected resolution at a half micron pixel size. To that end, the new optics characterized here address the need for high resolution over a large field of view as outlined above.

The measured resolution of approximately 1 µm is consistent with predictions in the literature based on the lens–scintillator combination we used. The most thorough analysis of the resolution of scintillator–lens pairs in X-ray imaging has been given by Koch *et al.* (1998 ▸). There they derive empirical formulas for the expected spatial resolution of a lens–scintillator pair accounting for both diffraction and defect of focus effects. The presented formulas and plots are for a specific emission wavelength (550 nm) and, importantly, assume no attenuation of X-rays in the scintillator, *i.e.* that emitted light is generated equally at all depths in the scintillator. For a relatively thick, 100 µm scintillator, like that used here, attenuation will be significant and most interactions will take place near the surface of the scintillator. At 20 keV, ∼50% of the interactions will take place in the first 25 µm. Evaluating the full width at half-maximum expression of Koch *et al.* for a 25 µm scintillator with a NA = 0.6, and assuming 520 nm rather than 550 nm light, yields a predicted value of 1.1 µm, quite similar to what we have measured in practice.

To accommodate larger samples, a second detector designed for a larger field of view is currently being designed to provide a 12 mm image circle. This new version will use a medium-format sensor with 151 million pixels in 14192 × 10640 format to provide a field of view of 10 mm × 7.5 mm. The new objective lens promises to provide the same switchable 0.6/0.75 NA, and diffraction-limited optical resolution of 0.5 µm at NA of 0.6 and maintains the same resolution at NA of 0.75. The effective pixel size will be 0.7 µm at the scintillator plane. Because the optical resolution scale is less than the effective pixel size, a three-step super-resolution acquisition process with 0.25 µm step size will be needed to fully realize the optical resolution. Once we are able to confirm these specifications, the combination of these two detector systems will provide additional versatility for different sample size versus resolution combinations.

As higher megapixel cameras enter the market and scanning capabilities continue to improve in both resolution and field of view, larger data sets will necessitate management of issues such as data storage, processing and curation. Access to high-performance computing, storage management, cloud-based data analytics, high-speed data transfer and local, laboratory-specific computing implementations will become necessary for data interrogation by general users of synchrotrons as well as for local facilities utilizing tabletop sources. In short, beyond image acquisition and reconstruction, assistance in data management, visualization and analysis will be needed for users or entities who wish to pursue this rapidly developing form of micro-CT, especially for community-level projects.

Along with our testing of new technologies in scintillator polishing, beam monitoring, and quantum efficiency improvements in high-resolution camera sensors, we anticipate significant improvements to synchrotron facilities utilizing our detector. In an ongoing collaboration with beamline 8.3.2 at the Berkeley ALS, we are working towards a permanent installation of our detector to make this high field-of-view, high-resolution imaging available to the full micro-CT user base. The larger field of view greatly increases the breadth of specimen types that can be imaged, including both biological and non-biological samples. The adaptability of such large-field, high-resolution optical systems to other synchrotron and non-synchrotron applications promises a broad potential range of benefits in the biomedical and physical sciences across the global micro-CT community.

## Supplementary Material

Figures S1 to S3. DOI: 10.1107/S160057752101287X/mo5248sup1.pdf
