# Supplementary material for: A wide-field micro-computed tomography detector: micron resolution at half-centimetre scale
Source: J Synchrotron Radiat. 2022 Jan 19;29(Pt 2):505–14. doi: 10.1107/S160057752101287X (PMC8900834; doi:10.1107/S160057752101287X)
Supplement: Supplementary file 1 [file s-29-00505-sup1.pdf]

JOURNAL OF
SYNCHROTRON
RADIATION

Volume 29 (2022)

Supporting information for article:

A Wide-Field Micro-Computed Tomography Detector: Micron Resolution at Half-centimeter Scale

Maksim A. Yakovlev, Daniel J. Vanselow, Mee Siing Ngu, Carolyn R. Zaino, Spencer R. Katz, Yifu Ding, Dula Parkinson, Steve Yuxin Wang, Khai Chung Ang, Patrick La Riviere and Keith C. Cheng

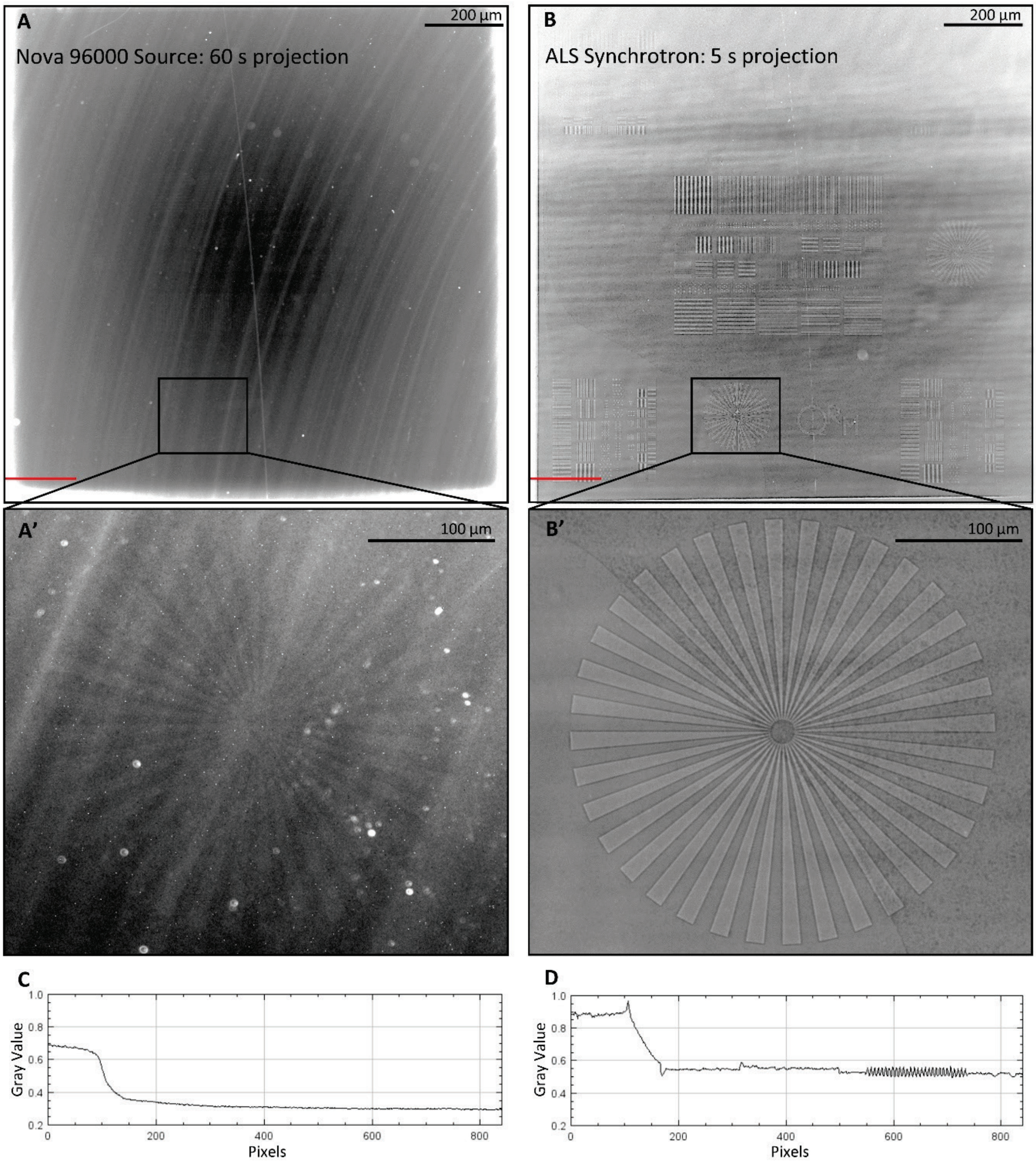

Figure S1 Projection comparison between the synchrotron beam and a commercially available x-ray source. Gain-corrected projections of the same QRM nanophantom are presented as acquired from an Oxford Nova 96000 source (A) and at ALS beamline 8.3.2. (B). These projections were acquired with the same detector system and scintillator, with the only difference being the x-ray source and exposure time. The phase effects of the monochromatic, parallel synchrotron beam highlighted phantom features etched into plastic, shown in the zoom-ins of A and B as the Siemens star wedges that start at 17 µm and narrow down to 1 µm at the tip (A', B'). These features are less

visible in the same area of the phantom when acquired using our local, cone-beam source (A'). The brilliance of the synchrotron beam also drastically reduces acquisition time per individual projection (150 ms for synchrotron and 60 s for the local source) to achieve comparable SNR, as shown in the line patterns (C,D) indicated by the red lines in A and B. Differences in the prominence of polish marks, scintillator spotting, and other background effects are due in part to beam flux and structure between synchrotron and local sources, as well as projections being taken at different positions on the scintillator.

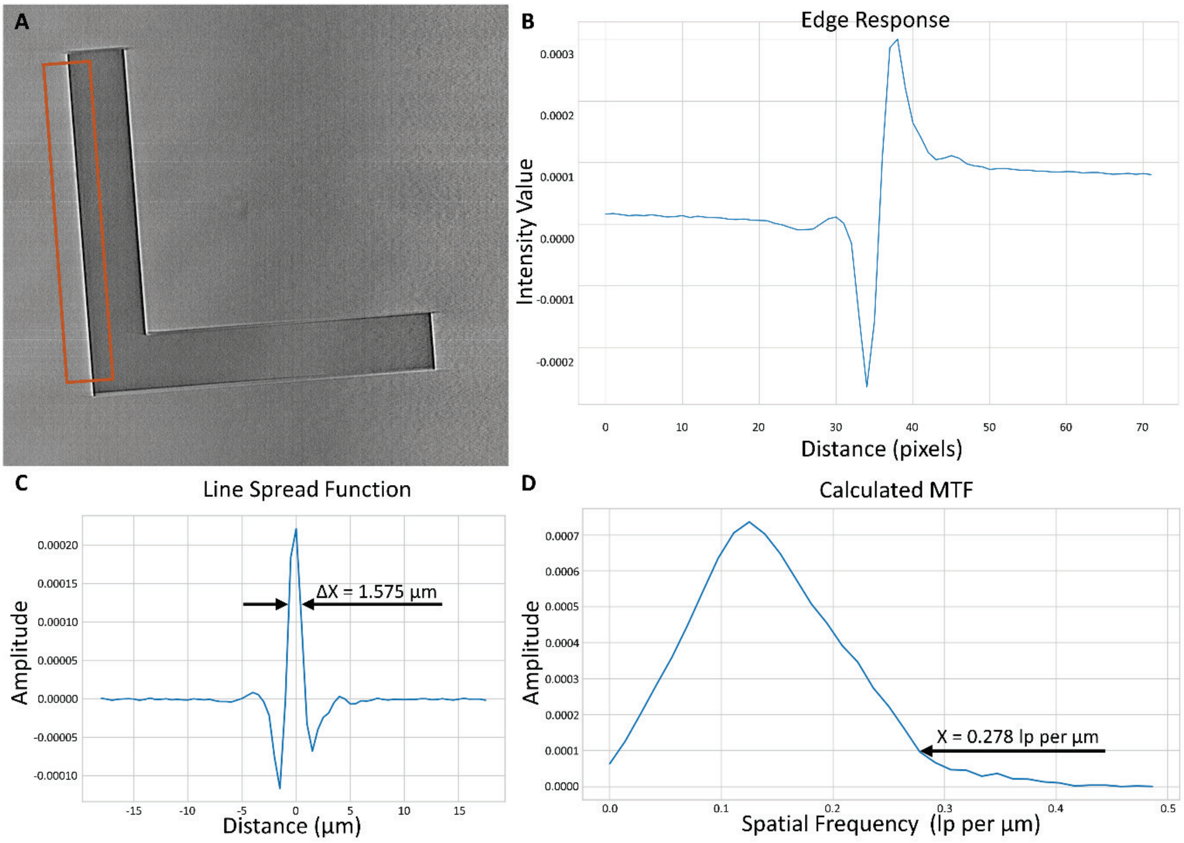

Figure S2 Slanted Edge Response and calculation of Modulation Transfer Function (MTF) in the reconstruction domain. The indicated area of the slanted L edge provided by the QRM bar pattern NANO phantom (A) was used to record the associated edge response (B), Line Spread Function (LSF) (C), and MTF (D). The effects of edge enhancement from the Berkeley ALS beam can be observed in the large intensity peaks of the edge response, the minima of the LSF, and decreased MTF at low spatial frequencies. The full width at half maximum amplitude of the LSF was recorded at 1.58 µm, with a 0.1 MTF amplitude at 0.28 line pairs per micron.

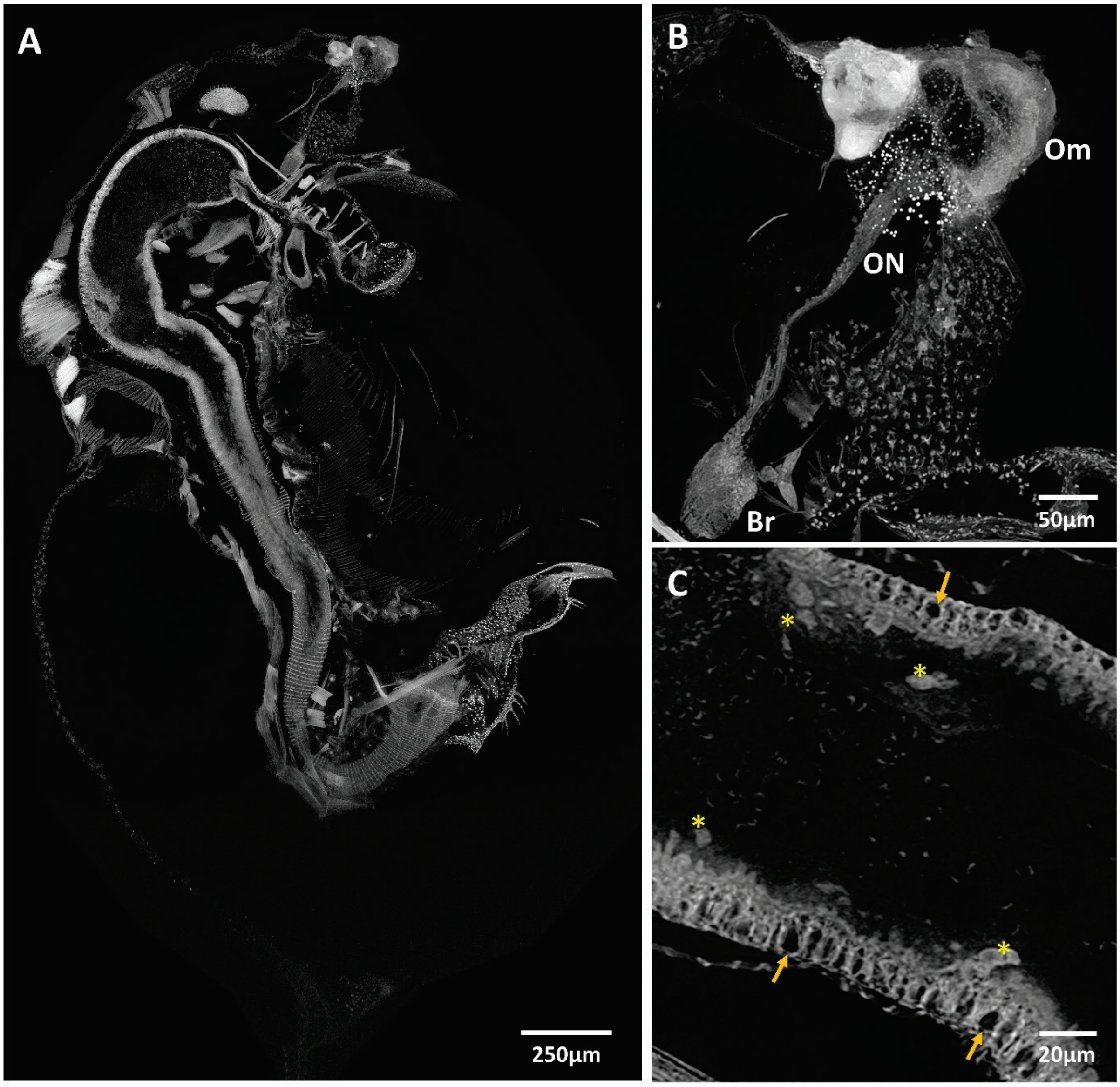

Figure S3 **An unhealthy *Daphnia magna* found in our wildtype population with its ommatidia developed outside the carapace (A).** Micro-CT imaging revealed deformities of the ommatidia (Om), longer optic nerves (ON) that connect directly to the brain (Br) but without a corresponding optic lobe (OL) (B). Whole-organism phenotyping also revealed cellular changes in the digestive tract, with vacuolation (orange arrows) and sloughing of gut epithelial cells (asterisks) (C). Panels A and B were generated from maximum intensity projections (MIP) of 50 slices, panel C from a MIP of 10 slices.
